# 

**Published:** 1893

**Authors:** 


					﻿HYGIENE HINTS FOR RAILWAY TRAVEL.
By MRS. E. A. MATTHEWS.
io cts. a copy.
SEPTEMBER-OCTOBER, 1893.
$1.00 per year.
WALES®
JOV^nalj
WEALT W
ESTABLISHED 1854
I/ql XL.
Wo. 9 & 10
OFFICE, 23 PARK ROW.
Entprpd Hr spponri>OlfitfR MAttftr at.
				

